# Association of air pollution and meteorological variables with the two waves of COVID-19 pandemic in Delhi: A critical analysis

**DOI:** 10.1016/j.heliyon.2021.e08468

**Published:** 2021-11-24

**Authors:** Abhishek Dutta, Gautam Dutta

**Affiliations:** a Department of Environmental Science, Faculty of Science, Chulalongkorn University, 254 Phayathai Road, Pathumwan, Bangkok 10330, Thailand; b Department of Management Studies, Indian Institute of Foreign Trade, 1583, Madurdaha, Kolkata, West Bengal 700100, India

**Keywords:** COVID-19 successive waves, Environmental variables, Air quality, Reproduction rate, Infection spreading trajectory

## Abstract

Various countries across the globe have been affected by different COVID-19 waves at different points in time and with varying levels of virulence. With the backdrop of the two COVID-19 waves that broke out in Delhi, this study examines the variations in the concentrations of criteria pollutants, air quality, and meteorological variables across the waves and their influence on COVID-19 morbidity/mortality. Descriptive statistics, violin plots, and Spearman rank correlation tests were employed to assess the variations in environmental parameters and investigate their associations with COVID-19 incidence under the two waves. The susceptible-infected-recovered model and multiple linear regression were used to assess the wave-wise basic reproduction number (R0) and infection spreading trajectory of the virus. Our results show that the first wave in Delhi had three successive peaks and valleys, and the first peak of the second wave was the tallest, indicating the severity of per-day infection cases. During the analysed period (April 2020 and April 2021), concentrations of criteria pollutants varied across the waves, and air pollution was substantially higher during the second wave. In addition, the results revealed that during the second wave, NO_2_ maintained a significant negative relationship with COVID-19 (cases per day), while SO_2_ had a negative relationship with COVID-19 (cumulative cases) during the first wave. Our results also show a significant positive association of O_3_ with COVID-19 deaths during the first wave and cumulative cases and deaths during the second wave. The study indicates that a higher relative humidity in Delhi had a negative relation with COVID-19 cumulative cases and mortality during the first wave. The study confirms that the estimated R0 was marginally different during the two waves, and the spread of COVID-19 new cases followed a cubic growth trajectory. The findings of this study provide valuable information for policymakers in handling COVID-19 waves in various cities.

## 1 Introduction

Countries worldwide are facing the resurgence of COVID-19 disease caused by the severe acute respiratory syndrome coronavirus 2 (SARS-COV-2) in the form of different waves at different points in time and with varying levels of virulence (Iftimie et al., 2021; Soriano et al., 2021; Jassat et al., 2021). Italy had an intense first wave during February–May 2020, and the second wave occurred August 2020–February 2021 with comparatively less impact than in other European countries (Bontempi, 2021; Coccia, 2021). Jalali et al. (2020) reported lower disease mortality during the second wave than the first wave in Iran, which struck during the relatively warm months (February–May). In contrast, Germany managed the first wave exceptionally well but suffered much in the second wave (Graichen, 2021). India's first wave of the COVID-19 pandemic began on 24 March 2020, and a more powerful second wave hit the country beginning February 2021 (Krishnakumar and Rana, 2020; Thiagarajan, 2021).

During the first wave, lockdown measures brought a significant reduction in air pollution across different cities worldwide (Rume and Didar-Ul Islam, 2020; Gautam, 2020; Zambrano-Monserrate et al., 2020; Venter et al., 2020; Liu et al., 2021). Kumari and Toshniwal (2020) reported reductions in PM_2.5_, PM_10_, and NO_2_ concentrations by 20–34%, 24–47%, and 32–64%, respectively, in 12 major world cities due to lockdown measures. Fu et al. (2020) noted similar findings for PM_2.5_, PM_10_, NO_2_, SO_2_, and CO concentrations in 20 major world cities due to the lockdown effect. Rathod et al. (2021) found reductions in three crucial air pollutants, namely, NO_2_, CO, and volatile organic compounds (VOCs), in Delhi by 50%, 37%, and 38%, respectively, during the lockdown period of 2020 as compared to the similar period in 2019. After the first wave, countries eased the stringent lockdown conditions considerably based on necessities and their capability to fight the pandemic. Yan et al. (2021) reported an average decline of more than 45% in stringency measures in 25 early COVID-19-affected countries compared to the strictest measures taken during the first wave. Singh et al. (2021) compared the air pollution of the top ten most COVID-19-affected countries under different lockdown stringency levels and indicated that ambient air pollution increased with the relaxation of lockdown measures without considering the seasonal effect.

Research during the first wave of the pandemic strongly supports the role of air pollution in the diffusion of SARS-CoV-2 viruses (Copat et al., 2020; Coccia, 2020; Prather et al., 2020; Fattorini and Regoli, 2020; Zoran et al., 2020). Furthermore, air pollutants are known to have a role in inducing respiratory toxicity; hence, they are likely to also aid respiratory illnesses such as COVID-19 (Comunian et al., 2020; Dutta and Jinsart, 2021a). Epidemiological studies also highlight the possible role of air pollution, in combination with meteorological variables such as low temperature and relative humidity (RH), in COVID-19 disease transmission (Lolli et al., 2020; Moriyama et al., 2020). Recently published review papers have also highlighted the role of air pollution and meteorological conditions in SARS-CoV-2 transmission (Andersen et al., 2021; Bourdrel et al., 2021; Tian et al., 2021; Zhao et al., 2021). Zoran et al. (2021) further identified the role of environmental variables during the multiple waves of COVID-19 in Madrid, Spain.

During the first wave, several research efforts were made across different world cities to capture spatiotemporal variations in air pollutants caused by the lockdown and the role they played in the spread of COVID-19. According to Dutta and Jinsart (2021b), approximately 16 studies were published on the first wave of the pandemic in Delhi, highlighting the lockdown effect on air quality and its relationship with COVID-19 disease spread. These studies proved to be immensely valuable for policymakers to understand the critical dynamics between air pollution and COVID-19 morbidity or mortality, thereby supporting the implementation of effective measures to control the spread of the pandemic. However, in the face of cities experiencing multiple waves, research efforts have been scarce in investigating the interplay between COVID-19 disease incidence and environmental conditions under different waves. It is essential to understand how changes in meteorological variables and air pollution interact with the spread of the pandemic during successive waves. Such findings will be of immense use to policymakers, guiding them in developing appropriate policy measures.

Therefore, the main objective of this study is to examine the variations in the concentrations of criteria pollutants, air quality, and meteorological variables across the waves and their influence on COVID-19 morbidity/mortality. To do this, the study first aims to understand the characteristics of the first and second waves of the COVID-19 disease outbreak in Delhi and compare the impact of the pandemic under the respective waves. Second, the study examines and compares the variations in criteria air pollutants, meteorological variables, and air quality indices over the matching periods during the two successive waves. Third, the study analyses and compares the COVID-19 morbidity/mortality of the waves with the environmental variables. Finally, as the city had a higher surge of COVID-19 positive cases and deaths during the second wave, the study examines and compares the two waves in Delhi in terms of their spread rates and trajectories to understand their similarities and differences. An understanding of how environmental variables influenced the COVID-19 morbidity/mortality of the two successive waves in the same city will shed more light on the link between meteorological variables and air quality factors and the COVID-19 pandemic.

We selected Delhi as the location for the study for three reasons: first, it is the world's most polluted city (IQAir Report, 2020; Kumar et al., 2020a); second, it witnessed the intense nature of the COVID-19 waves; and third, using the same city as the study location can neutralise the unmeasured confounding bias. To the best of our knowledge, this is the first study to assess the link between environmental variables and SARS-CoV-2 infection spread in the context of COVID-19 disease morbidity/mortality in the same city under two successive waves.

## 2 Methods

### 2.1 Study area

Delhi, located at 28.7041° N, 77.1025° E, is the capital city of India. The city covers a geographical area of 1484 sq. Km. Over time, Delhi has emerged as a significant city with respect to commerce, industry, medical services, and education, thereby attracting in-migration from the entire country. The city is projected to be the world's most populous by 2028 (UNDESA, 2018). The city is struggling to cope with its aggravating problem of air pollution and associated health hazards. Different sources like burning of fuels, industries, stubble burning of agricultural biomass residue in the neighbouring states, and vehicular movement are continuously pouring pollutants in the city air. In terms of vehicle stock, Delhi topped the Indian cities with 10.26 million vehicles during 2017 (Dutta and Jinsart, 2021c). According to the Köppen climate classification**,** Delhi's climate is extreme with five seasons. The summer is scorching with the maximum air temperature range of 43–46 °C, while winter is freezing with the minimum temperature range of 2–4 °C (Budhiraja et al., 2019).

### 2.2 Data sources

We collected data on the total number of COVID-19 cases, per day infection cases, mortality, and recovery for the study periods of April 2020 and April 2021, representing two successive pandemic waves, from the Delhi Government website (http://health.delhigovt.nic.in/), which is the source of all pandemic-related information in the city. The criteria air pollutants considered in this study included particulate matter with aerodynamic diameters of less than 2.5 and 10 μm (PM_2.5_, PM_10_), CO, NO_2_, SO_2_, and ozone (O_3_). Three important meteorological variables, namely temperature (T), RH, and wind speed (WS), were also considered. The daily mean concentration data for the criteria pollutants and meteorological variables for the study period were obtained from the offices of the Central Pollution Control Board (CPCB) and Indian Meteorological Department, Delhi, respectively. Table S1 lists the air pollution and weather station metadata.

### 2.3 Data analysis

During the study period, COVID-19 started spreading faster with the initiation of the two pandemic waves, and the local government marshalled different feasible or available methods to keep the outbreak under control. Delhi saw a very strict and effective lockdown during the first wave, while the second wave experienced a somewhat relaxed lockdown that was well supported by the initiation of a vaccination drive.

Descriptive statistics, such as mean, maximum (max), minimum (min), percentile, and interquartile range (IQR) were used to understand variations in the criteria pollutants and meteorological variables for the periods under study. Violin plots were used to infer the distribution patterns of environmental variables across the two waves of the pandemic. Due to the non-normal distribution of variables, Spearman's rank correlation test was used to examine the correlations between the criteria air pollutants and meteorological variables with COVID-19 spread. Spearman's rank correlation analyses were carried out using IBM SPSS Statistics 25 software, and correlation matrix plots were drawn using the ‘corrplot' package of R software.

Susceptible-infected-recovered (SIR) is a classic quantitative model for understanding the spread of an epidemic, and the model deals with three categories: suspected (S), infected (I), and recovered (R) portions of the population (N) of a city under epidemic attack. The basic SIR model was used to understand the characteristics of the infection cycle that prevailed during the two pandemic waves in Delhi. The following three linear differential equations frame the SIR model of infective disease spread (Hethcote, 2000):

(1) $$\;\frac{dS}{dt}=\;-\frac{\beta IS}{N},$$

(2) $$\frac{dI}{dt}=\;\frac{\beta IS}{N}-\;\gamma I,$$

(3) $$\frac{dR}{dt}=\gamma I,$$

where S(t)+I(t)+R(t)=N, and $S\left(0\right)=\;S_{0}\geq 0$, $\;I\left(0\right)=I_{0\;}\geq 0,$ and R(0)=R0≥0.

In the above three equations, β (infection transmission rate) and γ (recovery and removal rate) are fundamental parameters governing the infection spread dynamics. The move from susceptibility to infection (S to I) of the population will depend on β, while infection to recovery or death (I to R) will depend on γ. The above three differential Eqs. 1, 2, and 3 must be solved for S, I, and R numerically to determine the values of the parameters β and γ. The R software package called 'deSolve' was used to solve the ordinary differential equation systems using Runge-Kutta methods and determine parameters β and γ and the R0 (basic reproduction number) from Eq. (4) as shown below.

(4) $$R0=\frac{\beta }{\gamma }$$

The R0 value is vital for understanding the disease severity. If R0 is less than 1 (R0 < 1), it indicates a controlled epidemic situation in which one infected person will be infecting fewer than one individual on average. Similarly, if R0 is greater than one (R0 > 1), the epidemic is genuinely serious, as each infected individual will be able to infect many more individuals in their vicinity. Satsuma et al. (2004) found that the SIR model is appropriate for describing a well-localised epidemic outburst and, hence, can be used at the city level, as in the case of Delhi**.**

Regression models were used to trace the infection-spreading trajectory in successive waves of COVID-19. The models used the total cumulative cases for the study periods as the independent variables and the time horizon as the dependent variables to find the best-fit curves of the respective waves. The best-fit curves will be the infection-spreading trajectories of the COVID-19 waves in Delhi. We fitted 11 classical regression models using IBM-SPSS version 25 (Table S2). The R^2^ statistics of the regression models, as well as the respective standard errors, are considered to yield the best-fit curve.

## 3 Results and discussions

### 3.1 Spread of COVID-19 in Delhi: first wave vs second wave

**Figure 1:**
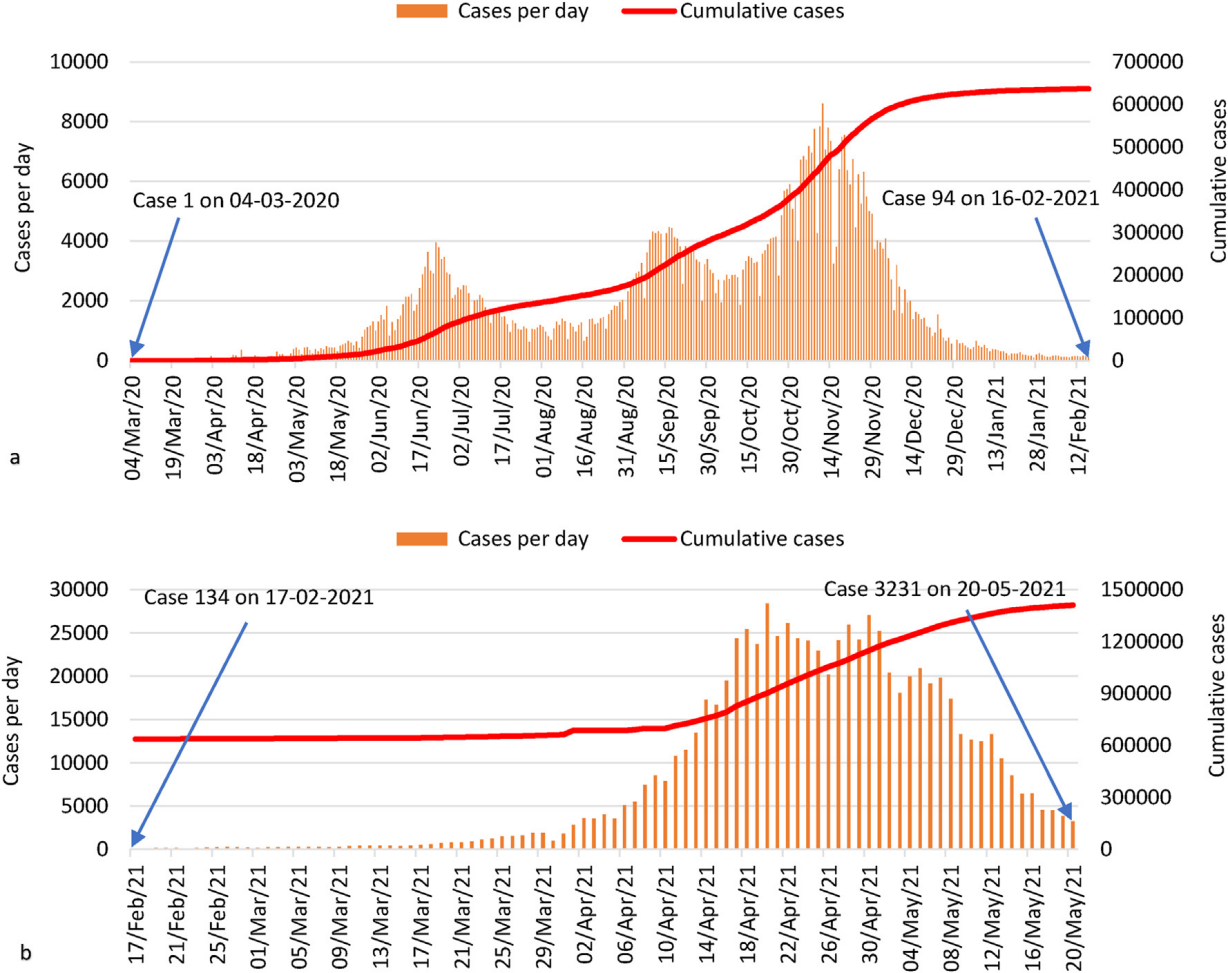
COVID-19 per day and cumulative cases during (a) first wave (1 March 2020 to 16 February 2021) (b) second wave (17 February 2021 onwards) in Delhi.

There is no clear definition of what constitutes a COVID-19 wave. However, it is evident from past infectious diseases that the outbreak and subsequent disease spread follows a waveform pattern with peaks and valleys. Notably, there is a lull period of infection spread, signalling the end of one wave but retaining the indication of the beginning of the next wave (Wagner, 2020; Zhang et al., 2021). As shown in Figure 1a, the spread of COVID-19 in Delhi followed a waveform pattern with peaks and valleys. The first COVID-19 wave began in Delhi on 4 March 2020, when the first COVID-19 case was detected in the city. The number of SARS-CoV-2 infection cases increased rapidly, and the first peak of the first COVID-19 wave in the city was reached on 23 June 2020, with 3947 cases per day, as shown in Figure 1a. The second peak came after three months, when approximately 4473 cases were reported on 16 September 2020. The third and the tallest peak was reached in the winter season, with 8593 new infected cases reported on 11 November 2020. Figure 1a also indicates that the successive peak heights had an increasing trend, and the peak that occurred during the winter months of Delhi had the greatest height. This finding supports the hypothesis that an easier spread of COVID-19 is possible in winter (Chen et al., 2021). After the third peak, the infected cases reported per day fell rapidly, and by the beginning of the new year 2021, the daily new infections fell below 500. Finally, Delhi heaved a sigh of relief when the number of infected cases registered per day was as low as 94 on 16 February 2021, thereby marking the end of the first wave of the pandemic. As shown in Figure 1a, the first wave lasted nearly a year, that is, from 4 March 2020 to 16 February 2021, with three successive peaks and valleys in between.

Figure 1b depicts the characteristics of the COVID-19 s wave in Delhi. Figure 1b shows that from 17 February to 22 March 2021 new infection cases per day were, on average, 343. Figure 1b shows that starting from 23 March 2021, new cases jumped to a four-digit figure per day, signalling the beginning of the second wave. On 20 April 2021, the second wave reached its first peak with a new city record of 28395 new cases in a single day. A comparison between Figure 1a and b reveals that the second wave of COVID-19 was harsher than the first wave with regard to the disease spread. The local administration used measures such as herd immunity building through vaccination and a partial to complete lockdown to bring the new infection cases down to approximately 4000 cases per day by mid-May 2021, as shown in Figure 1b.

**Figure 2:**
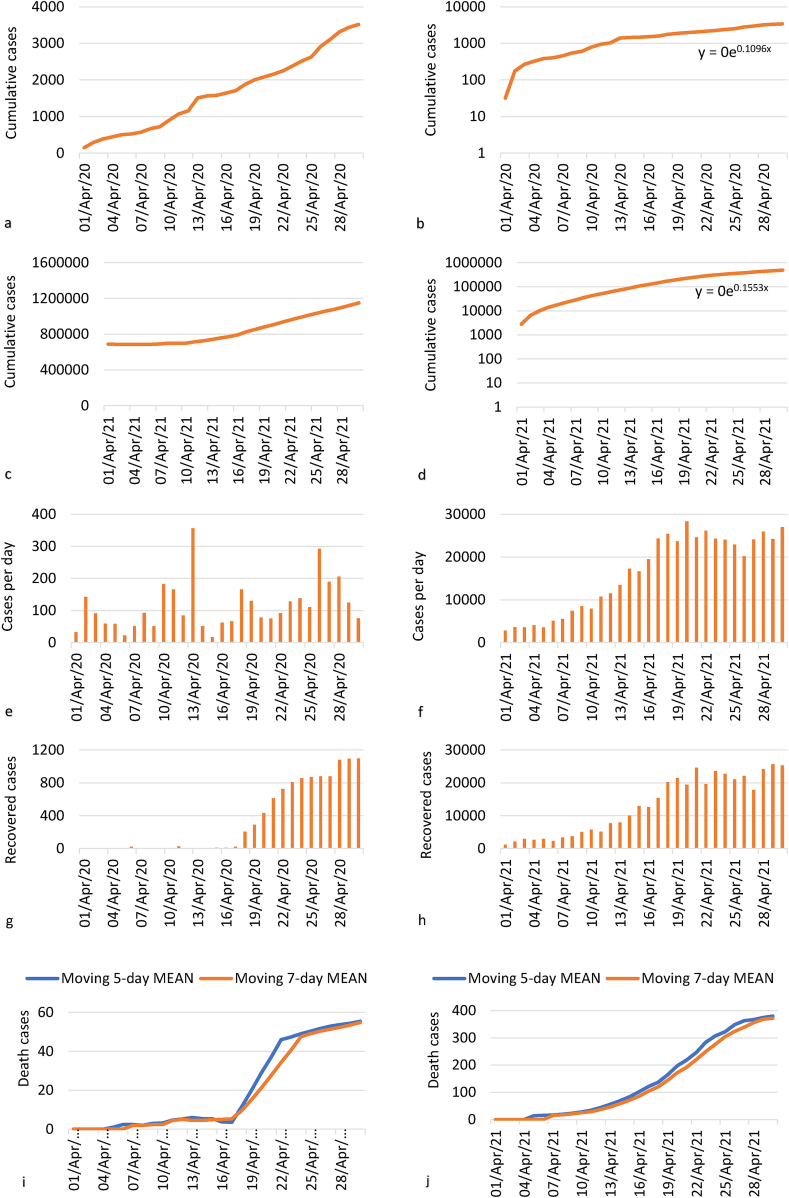
Incidence of COVID-19 cases in Delhi (a) daily cumulative, April 2020 (b) daily cumulative, April 2021 (c) daily cumulative, April 2020 (log scale) (d) daily cumulative, April 2021 (log scale) (e) daily incremental incidence, April 2020 (f) daily incremental incidence, April 2021 (g) recovery cases per day, April 2020 (h) recovery cases per day, April 2021 (i) daily deaths, April 2020 (moving mean), (j) daily deaths April 2021 (moving mean).

Table 1 compares COVID-19 cases in Delhi across two sample periods: April 2020 of the first wave and April 2021 of the second wave. The April 2020 data set has more variability in COVID-19 infection cases reported per day than does April 2021, as revealed by the higher coefficient of variation shown in Table 1. Figure 2a and b show the time-series graphs of the daily cumulative incidence of confirmed COVID-19 cases for Delhi over the two-sample periods on a linear scale. The linear graph of April 2020 shows a steady increase in cases during the period, and the graph of April 2021 indicates a slow increase for the first six days, followed by rapid growth. Figure 2c and d display the same data, that is, the daily cumulative incidence of confirmed COVID-19 cases for Delhi in April 2020 and April 2021, respectively, on a log-linear scale. The log-linear scale plots of Figure 2c and d show the cumulative incidence of the pandemic in the city in a more interpretative way (Sevi et al., 2020). First, Figure 2c and d indicate that the cumulative incidence of COVID-19 grew exponentially in Delhi in both waves. Second, the fitted line estimates the doubling time of COVID-19 for both April 2020 and April 2021. The slope of the fitted line is 0.1096 log cases per day in April 2020 and 0.1553 log cases per day for April 2021. Therefore, the doubling time for COVID-19 in April 2020 was 6.32 days and that for April 2021 was 4.46 days. This indicates that COVID-19 spread faster during the second wave than during the first wave.

**Table 1:** Comparison of COVID-19 cases in Delhi across two sample periods (April 2020 and 2021).

City	Total cases of infection		Highest cases reported in a day		Lowest cases reported in a day		Mean		SD		Co-efficient of variation (%)	
	April 2020	April 2021	April 2020	April 2021	April 2020	April 2021	April 2020	April 2021	April 2020	April 2021	April 2020	April 2021
Delhi	3515	486903	356	28395	17	2790	113	16230	76.8	9057.3	68	55.8

Figure 2e and f show the daily incremental incidence of COVID-19 cases for April 2020 and April 2021, respectively. It can be observed from Figure 2e and f that April 2021 was much farther ahead of April 2020 regarding the number of infections per day, as the average cases of 16230 for April 2020 were much greater than those of only 113 for April 2020. Figure 2g and h compare the COVID-19 recovery cases per day, and it can be seen that during April 2020, the average recovery cases per day was 331, while in April 2021, it was much higher at 13042 cases per day. This reflects that during the second wave, both the incidence and recovery of per day COVID-19 cases were higher than those in the first wave.

Figure 2i and j show the moving average daily deaths during April 2020 and April 2021, respectively. The mortality curves provide critical visual information about mortality due to COVID-19 cases during the two waves in Delhi. During April 2020, the COVID-19 mortality increased abruptly after mid-month and then flattened to some extent. In contrast, during April 2021, mortality started showing an almost exponential pattern from the first week.

### 3.2 Distribution of air pollutants, meteorological variables and COVID-19 cases: first wave vs second wave

Table 2 provides the descriptive statistics of the criteria air pollutants, meteorological variables, and COVID-19 cases that prevailed in Delhi between April 2020 and April 2021. Both PM_2.5_ and PM_10_ increased by 78.1% (37.3–66.4 μg m^−3^) and 106.1% (95.8–197.5 μg m^−3^), respectively, during April 2021 to April 2020. Similarly, the mean NO_2_ and CO concentrations increased by approximately 229.4% and 77.1%, respectively, in April 2021 compared to the corresponding month of the previous year when COVID-19 infections first started in Delhi. The increases in PM_10_, PM_2.5_, NO_2_, and CO concentrations were primarily due to a substantial increase in anthropogenic activities during the second wave in the city (Anhäuser and Farrow, 2021; Gautam et al., 2021).

**Table 2:** Distribution of criteria pollutants, meteorological variables and COVID-19 cases, Delhi, April 2020 and April 2021(Sample size, N = 30).

Variables	Year	Mean ± SD	Max	Min	Percentile			IQR
					25th	50th	75th	
T (°C)	2020	28.4 ± 2.6	32.7	23.8	26.5	0.6	30.5	4.0
	2021	29.3 ± 2.6	35.1	25.8	27.2	29.1	31.0	3.8
RH (%)	2020	59.9 ± 13.9	85.0	25.9	49.7	59.1	71.8	22.1
	2021	56.1 ± 9.5	76.4	39.7	49.8	57.5	62.4	12.6
WS (m s^−1^)	2020	4 ± 1.1	7	2	2.9	3.4	4.1	1.2
	2021	4 ± 1.0	6	1	3.4	3.7	4.2	0.8
PM_2.5_ (μg m^−3^)	2020	37.3 ± 13.1	58.8	18.3	24.7	35.1	47.9	23.2
	2021	66.4 ± 32	143.3	27.8	44.1	60.4	78.1	34
PM_10_ (μg m^−3^)	2020	95.8 ± 32.7	190.7	51.1	74.4	91.4	109.1	34.7
	2021	197.5 ± 113.2	337.7	86.7	136.2	193.4	249.4	113.2
NO_2_ (μg m^−3^)	2020	16.1 ± 6	30.5	6.5	11.5	15.1	21.4	9.9
	2021	53 ± 17.5	92.1	23.9	39.7	53.4	66.5	26.9
SO_2_ (μg m^−3^)	2020	23.6 ± 4	36.5	17.3	21.4	23.2	25.6	4.2
	2021	24 ± 5.8	35.6	14.1	19.7	23.4	27.9	8.2
CO (μg m^−3^)	2020	0.5 ± 0.2	1	0.2	0.3	0.4	0.6	0.3
	2021	0.9 ± 0.4	1.5	0.3	0.5	0.8	1.2	0.7
O_3_ (μg m^−3^)	2020	62 ± 9.9	80	39	55.7	61.9	67.4	11.7
	2021	48 ± 11.2	62	18	39.9	50.2	57.4	17.5
COVID-19 cases/day	2020	113 ± 76.8	356	17	59.8	91.5	141.8	82
	2021	16230 ± 9057.3	28395	2790	7552	18384	24307	16755

However, it is worth mentioning that the SO_2_ concentration of 23.6 μg m^−3^ during April 2020 remained almost identical (24 μg m^−3^) to that of April 2021. Substantial SO_2_ in ambient air during the first wave and a nearly unchanged status during the second wave reflects its lower dependence on local sources for generation (Dutta and Jinsart, 2021b). Table 2 also indicates that the mean O_3_ concentrations decreased from 62 μg m^−3^ in April 2020 to 48 μg m^−3^ in April 2021, resulting in a 22.6% decrease. Delhi had a notable increase in ground-level O_3_ concentrations during the lockdown period of April 2020, in contrast to other air pollutants (Garg et al., 2021). A substantial reduction in NOx in the ambient air due to the lockdown effect led to a lower titration of O_3_ by NO (Han et al., 2011; Rathod et al., 2021). Table 2 also indicates that during April 2021, the mean temperature was marginally higher (29.3 °C) than in April 2020 (28.4 °C). However, the RH was lower (56.1%) in April 2021 than in April 2020 (59.9 %). There was almost no change in the wind speed, which remained at 4 m s^−1^ during both waves. Sahoo et al. (2021) also reported very minor variations in wind speed during the pre-and lockdown periods in Delhi. The maximum number of new COVID-19 cases per day during April 2020 was 356, which increased to 28395 during April 2021, indicating a faster spread of infection during the second wave (Asrani et al., 2021).

**Figure 3:**
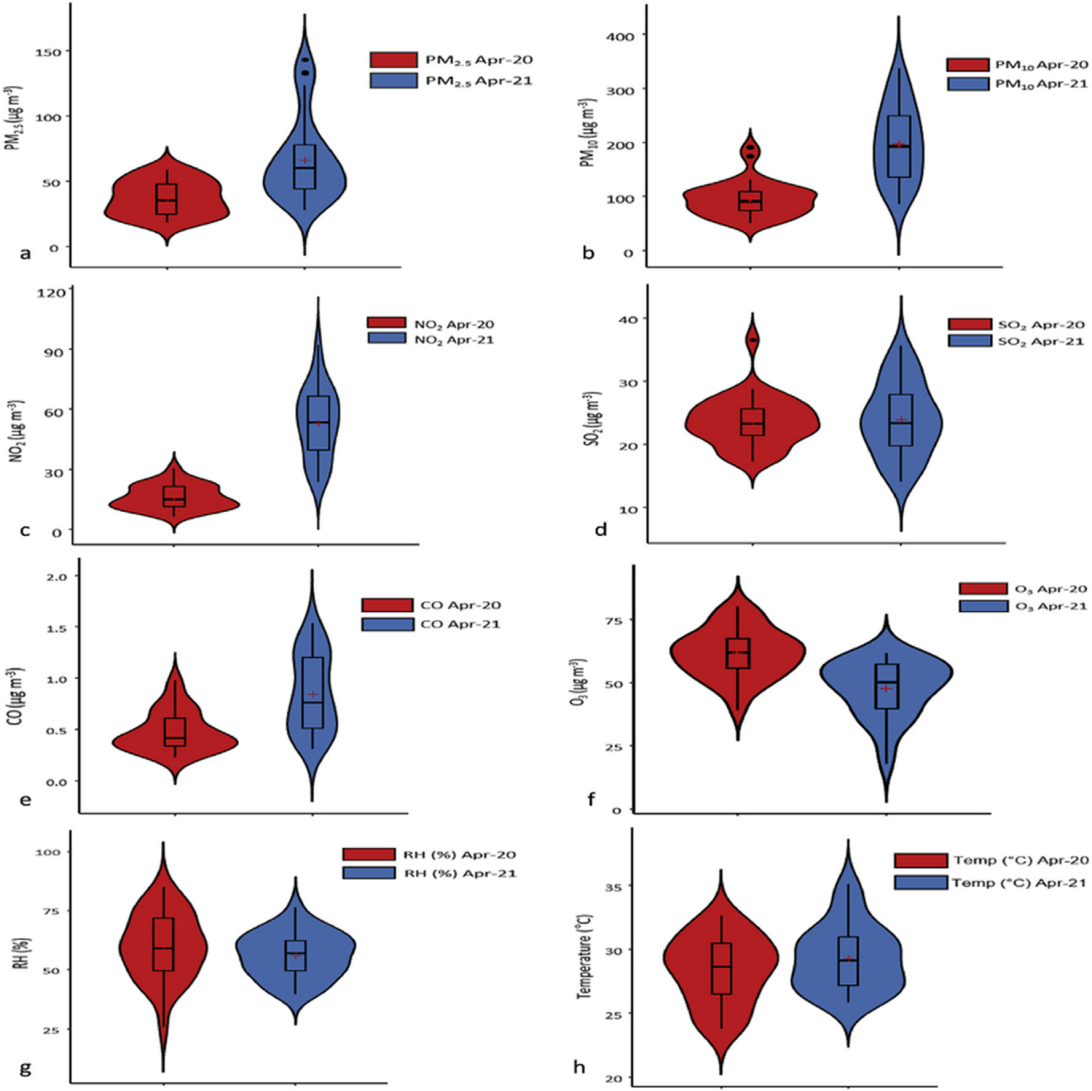
Violin plots of (a) PM_2.5_ (μg m^−3^)_,_ (b) PM_10_ (μg m^−3^), (c) NO_2_ (μg m^−3^), (d) SO_2_ (μg m^−3^), (e) CO (μg m^−3^), (f) O_3_ (μg m^−3^), (g) Relative humidity (%), (h) Temperature (ºC).

Figure 3a–h provide the violin plots of the critical criteria pollutants and selected meteorological variables for the city of Delhi. In the violin plots, the rectangle in the center denotes the IQR of the data distribution, while the central cross represents the median value, and the whiskers show 1.5 × IQR for the rest of the data. The violin plots of PM_2.5_, PM_10_, and NO_2_ indicate that median values of the respective data distributions were lower during April 2020 than April 2021. The violin plot of SO_2_ suggests that the April 2020 median value (23.2 μg m^−3^) was marginally lower than that for April 2021 (23.4 μg m^−3^), and the SO_2_ pollution was more clustered around the median during April 2020, as shown by the width of the violin, unlike in April 2021. Two meaningful inferences can be made from the SO_2_ violin plots. First, during April 2020, SO_2_ concentration distributions were comparatively more stable than those of April 2021, and second, COVID-19 prevention measure differences during the two successive waves did not significantly alter the pollutant's concentration in the air.

Violin plots of O_3_ indicate that the pollutant's median value was considerably higher during April 2020 than April 2021, with the concentration distribution more clustered around the median in comparison. It is apparent from the violin plots of RH that there was a marginal difference in the median values of the variables for April 2020 and April 2021. However, a smaller IQR of RH during April 2021 indicates values that were less spread out from the median than in April 2020. Regarding temperature, little difference was seen in both the range and dispersion pattern of 2021 compared with that of 2020. In general, for the April 2021 situation in Delhi, the violins of PM_2.5_, PM_10_, NO_2_, SO_2_, CO, and O_3_ had larger IQRs, suggesting that their values were more spread out from the median than in April 2020.

### 3.3 Variations in concentrations of criteria air pollutants: first wave vs second wave

**Figure 4:**
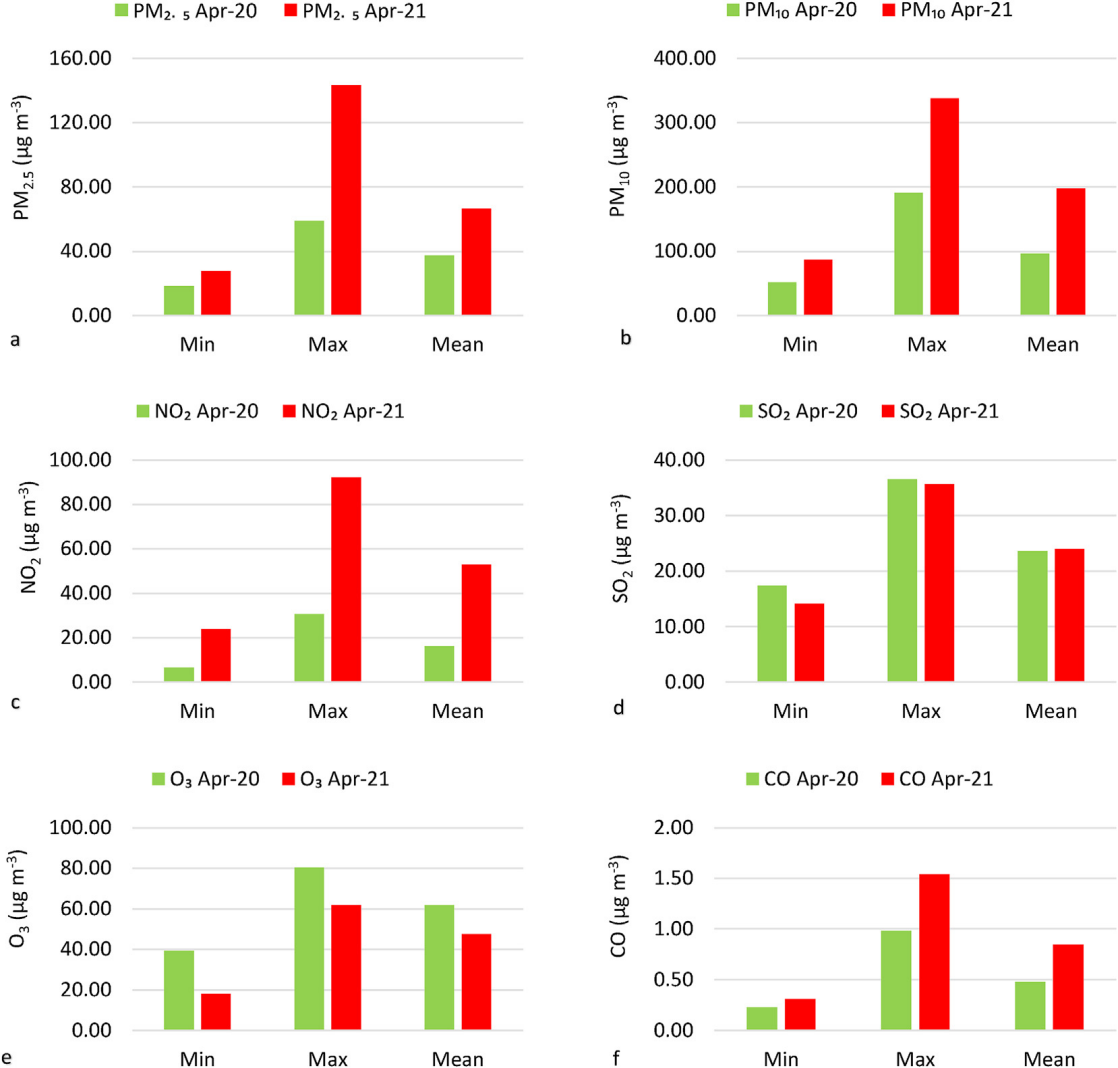
The mean (a) PM_2.5_ (b) PM_10_ (c) NO_2_ (d) SO_2_ (e) O_3_ (f) CO concentrations of Delhi during April 2020 and April 2021 of COVID-19 outbreak.

Figure 4a–f show the concentration variations for criteria pollutants across the two sample periods of April 2020 and April 2021 in Delhi. Figure 4a indicates that the city's mean, minimum, and maximum PM_2.5_ concentrations were 37.3, 18.3, and 27.8 μg m^−3^, respectively during April 2020. However, the PM_2.5_ mean, minimum, and maximum concentrations changed to 66.4, 58.8, and 142.3 μg m^−3^, respectively during April 2021. The sharp reduction in the PM_2.5_ level during April 2020 was made possible by the complete lockdown leading to unprecedented reductions in anthropogenic activities in the city from the morning of 23 March 2020 (Dhaka et al., 2020; Mahato et al., 2020). According to Sharma and Mandal (2017), almost 46% of PM_2.5_ build-up in Delhi comes from secondary aerosols and soil dust, and the rest is constituted by vehicle emissions, fossil fuel burning, biomass burning, and industrial emissions during normal times. Therefore, during April 2020, when Delhi had a stringent lockdown with a complete closure of the transport and industrial sectors, it witnessed the lowest count of particulate matter in the recent history of air pollution. However, during the second wave (April 2021), Delhi did not resort to a complete lockdown, and hence the mean PM_2.5_ level shot up by more than 78.1% of the first wave (April 2020).

Figure 4b indicates that because of the relaxed lockdown associated with the resumption of construction and industrial production activities during the second wave, the PM_10_ concentration increased more than 106.1% from April 2021 to April 2020. Figure 4c indicates that the mean NO_2_ concentration increased by 229.4% in April 2021 compared to April 2020. Industrial production and traffic activities continued almost normally, and a fear of the second wave of the COVID-19 epidemic was yet to hit the city. Figure 4d shows that the SO_2_ mean concentration level was only by 2% higher in April 2021 than in April 2020 when the city had the strictest lockdown with the road transport sector almost under a shutdown status. Apart from natural sources, SO_2_ can be generated from burning sulphur-rich fossil fuel in the highly urbanised city environment. Delhi has thermal power generating stations on its periphery, which act as a source of SO_2_ for the city. In addition, biomass burning in the bordering states historically reinforced the SO_2_ level in the city along with some contribution from local industries (Goyal and Sidhartha, 2002; Datta et al., 2010). Therefore, it is clear that a substantial part of the SO_2_ build-up must have been from non-local sources travelling from distant places and stubble burning in the neighbouring states.

Table 2 and Figure 4e indicate the differences in O_3_ concentration during April 2021 and April 2020. Delhi usually suffers from a high ground-level O_3_ concentration, which is regarded as an important reason for the rise of respiratory diseases in the city. Figure 4e reveals that the strict lockdown during April 2020 could not provide relief to the city dwellers. However, when the lockdown was significantly relaxed in April 2021, the mean ground-level O_3_ concentration decreased by 29.3% but still had a strong presence in the ambient air. One of the important sources of CO in Delhi is the road transport sector and its motor vehicles. CO is also contributed by other local sources, such as the use of diesel generators in industries, thermal power plants, and domestic or commercial cooking using coal or petroleum products. The usual high level of CO concentration in the city atmosphere is also reinforced through crop burning in neighbouring states (Tyagi et al., 2016). Figure 4f reveals that the mean CO concentration in Delhi during April 2021 was 77.1% higher than that in April 2020. The large increase in CO concentration proves two important points: first, a large portion of CO originates from the running of motor vehicles; two, the strict lockdown of April 2020 crippled road transport during April 2020 but not in April 2021.

### 3.4 Air quality index (AQI) distribution: first wave vs second wave

We calculated the AQI values of Delhi for April 2021 and April 2020 and plotted them in Figure S1a–b to understand how the AQI values impacted the city under the two waves of the pandemic outbreak. Figure S1a shows that the mean AQI value was 93, with a minimum of 51 and a maximum of 160 in April 2020. It can be observed from Figure S1b, the AQI values increased substantially during April 2021, with a mean of 177, a minimum of 87, and a maximum of 318. Therefore, it is obvious that different patterns of AQI values resulted as the constituent pollutants changed quantitatively under the two different regimes of lockdown measures during April 2020 and April 2021. With the change in AQI values, the AQI classes that prevailed during April 2020 looked better and healthy as 53.3% of the time during the month, the class II (satisfactory) environment prevailed, and the remaining 46.67% of the time, it was class III (moderate), as shown in Figure S2a. The distribution of AQI classes in Delhi during April 2021 indicated the worsening air pollution status of the city. In other words, during April 2021, 26.7% of the time, Delhi experienced poor and very poor AQI classes. In addition, the satisfactory class II was reduced to only 6.7% during April 2021, as shown in Figure S2b. Therefore, this implies a marked difference in Delhi's air quality status during the two waves of the COVID-19 period.

### 3.5 COVID-19 waves and their association with environmental parameters

**Figure 5:**
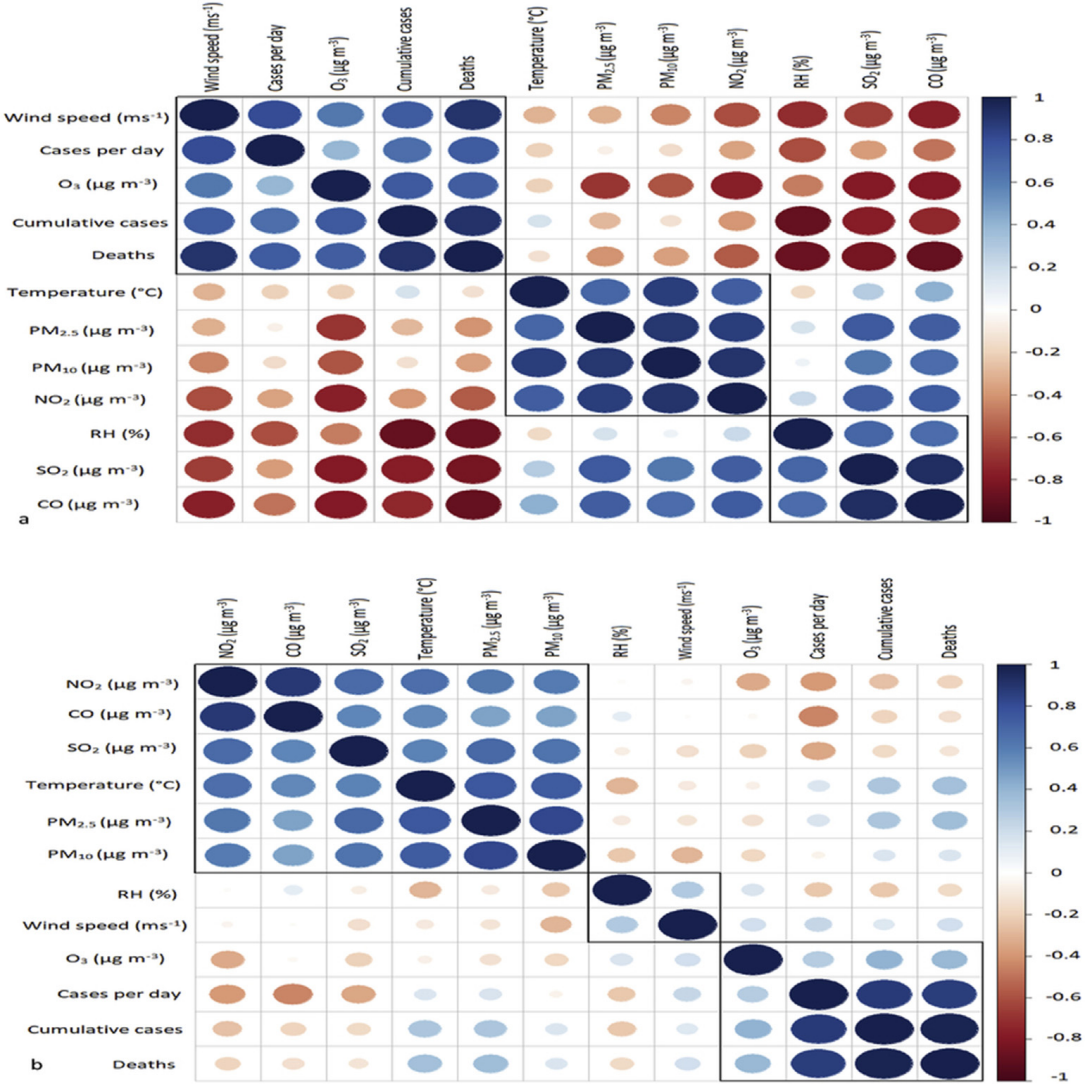
Spearman correlation matrix, (a) April 2020 and (b) April 2021 generated using R program. Colour code, Blue: positive correlations, Red: negative correlations, White: no correlation.

Spearman correlation tests were performed to understand the degree of association of criteria pollutants and meteorological variables with the cases of COVID-19 during the two waves of the pandemic in Delhi (Table 3 and Figure 5a and b). Table 3 shows no association between PM pollution (PM_2.5_ and PM_10_) and COVID-19 in Delhi during either pandemic wave. As indicated in Figure S3a and b, the mean RH level of Delhi only varied marginally across the waves; however, the COVID-19 cases per day varied widely. The Spearman correlation coefficients, shown in Table 3, indicate a significant negative correlation between the RH (mean = 60%) and daily COVID-19 new cases (r = −0.578; p < 0.01) and mortality (r = −0.501; p < 0.05) during the first wave. During the second wave, when Delhi had RH only marginally lower (RH = 56.1%), COVID-19 cases showed a negative correlation without reaching a significant level. Previous studies supported a low RH, approximately below 50%, to be positively correlated with COVID-19 cases and vice versa (Zoran et al., 2020). Therefore, this study outcome strengthens the hypothesis that a RH above 50% has a negative relationship with the spread of the novel coronavirus disease (Mecenas et al., 2020).

**Table 3:** Spearman correlation coefficients: Environmental factors and COVID-19 cases during two waves.

Variables	April 2020 1st wave			April 2021 2nd wave		
	Cases per day	Cumulative cases	Deaths	Cases per day	Cumulative cases	Deaths
PM_2.5_ (μg m^−3^)	−0.005	0.126	0.111	0.152	0.313	0.357
PM_10_ (μg m^−3^)	−0.012	0.261	0.153	−0.052	0.146	0.149
NO_2_ (μg m^−3^)	−0.168	0.093	0.004	−.389∗	−0.252	−0.187
SO_2_ (μg m^−3^)	−0.267	−0.376∗	−0.320	−0.340	−0.165	−0.118
CO (μg m^−3^)	−0.378∗	−0.629∗∗	−0.679∗∗	−.448∗	−0.187	−0.148
O_3_ (μg m^−3^)	0.102	0.326	0.458∗	0.282	.401∗	.383∗
Temperature (°C)	−0.043	0.502∗∗	0.354	0.148	0.314	0.340
RH (%)	−0.291	−0.578∗	−0.501∗∗	−0.235	−0.240	−0.168
Wind speed (ms^−1^)	0.202	0.182	0.254	0.223	0.136	0.186

∗∗, ∗ signifies significance at 5% and 1%.

The mean temperature that prevailed during the first wave (28.38 °C) was marginally lower than that during the second wave of the pandemic. Table 3, Figure S3c, and d show that the temperature was strongly and positively correlated with COVID-19 total cases (r = 0.502; p < 0.05) during the first wave. Previous studies have indicated that low temperatures in the range of approximately 2 °C–17 °C facilitated the virus spread (Guo et al., 2019; Roy, 2020). The facilitating role of temperature in higher temperature situations (mean 28–29 °C) found in this study is a departure from previous findings. We consider the deviation primarily due to the confounding COVID-19 super-spreading event reported in Delhi during March–April 2020 (Kumar et al., 2020b).

Table 3 shows that CO and COVID-19 cases had significant negative correlations during the first wave; for new cases (r = −0.378; p < 0.01), total cases (r = −0.629; p < 0.05), and deaths (r = −0.679; p < 0.05). However, for the second wave, only COVID-19 cases per day showed a significant negative correlation (r = −0.448; p < 0.01). Previous literature supports the positive role of CO in ambient air as the driver of COVID-19 new cases and deaths (Zhu et al., 2020). However, a recent study of the COVID-19 spread in London showed that CO was positively correlated with the number of new infection cases but did not reach the significance level (Meo et al., 2021). Therefore, more studies are required to establish the exact role of CO in ambient air in the spread of the novel coronavirus.

Table 3, Figure S3e, and f indicate that COVID-19 cases per day had a significant negative correlation with NO_2_ only during the second wave. This finding is not in agreement with the results of previous studies. Ogen (2020), in a multicounty study during the onset of the first wave, hypothesised a positive association between high and persistent NO_2_ exposure in the city population and high COVID-19 fatality rates because of the ability of NO_2_ to induce additional distress in the human respiratory system.

Table 3 and Figure S3e and f reveal that substantially high O_3_ concentrations in the ambient air during the first wave were positively and significantly correlated with COVID-19 mortality (r = 0.458; p < 0.05). During the second wave, when the O_3_ concentration was 29.34% lower than during the first wave, O_3_ still maintained a significantly positive correlation with COVID-19 cumulative cases (r = 0.401; p < 0.05) as well as mortality (r = 0.383; p < 0.05). This finding is in line with those of several previous studies. Fattorini and Regoli (2020) found a strong positive correlation between chronic exposure to ground-level O_3_ concentration and the COVID-19 spread in 71 Italian provinces. Tripepi et al. (2021) found that O_3_ in ambient air facilitates the spatial spread of the infection. Lim et al. (2021) also found strong evidence of the influence of O_3_ on the spread of COVID-19 in the Republic of Korea.

The negative correlation of NO_2_ found in this study with the COVID-19 cases per day during the second pandemic wave indicates an indirect role of the pollutant in influencing the spread of COVID-19 by influencing the O_3_ concentration in the ambient city air. Zoran et al. (2020) found similar findings in Milan, Italy, during the first wave of the pandemic, with daily new COVID-19 cases positively correlated with O_3_ and negatively correlated with NO_2_ concentrations.

This study found a significant negative correlation (r = −0.376; p < 0.01) between COVID-19 total cases and the concentrations of SO_2_ in the ambient air during the first wave (Table 3 and Figure 5a and b). Previous studies have reported contradictory findings regarding the correlation between the presence of SO_2_ in the air and COVID-19 cases. Zhu et al. (2020), in their study of 120 Chinese cities during the first wave, found a negative correlation between SO_2_ and daily COVID-19 confirmed cases. However, another study of 303 Chinese cities could not establish any statistically significant correlation between SO_2_ and COVID-19 daily cases during the onset of the first wave (Ran et al., 2020). The findings of this study align with the former, which indicates a possible virucidal role of SO_2_ in the spread of COVID-19. However, more research needs to be done as in this study SO_2_ did not significantly correlate with the COVID-19 spread, although it had almost the same concentration during the second wave.

Table 3, Figure S3k, and l indicate no significant association between wind speed and the spread of COVID-19 during both pandemic waves in the city. Several past studies have reported a positive association between wind speed and COVID-19 new cases (Mehmet, 2020; Dbouk and Drikakis, 2020; Aidoo et al., 2021). Therefore, more studies are required to establish the relationship between wind speed and COVID-19 spread.

### 3.6 The SIR model and R0: first wave vs second wave

For both the first wave (April 2020) and the second wave (April 2021), we set up and solved the systems of ordinary differential equations of the SIR model at the city level described in the methodology section 2.3. The cumulative number of cases for the 30 days of April 2020 and April 2021 was utilised to estimate the transmission rate (β) and recovery plus removal rate (γ) and then ascertain R0 of the SIR model. To fit the data in the SIR model and solve three differential equations, we used the function 'ode' from the 'deSolve' package available in the repository (CRAN) for R packages and then optimised with the 'optim' function in R. Table 4 shows the SIR model output corresponding to both COVID-19 waves in Delhi. The R0 value was greater than 1 in both waves, indicating that the infection rate was greater than the recovery rate. Therefore, the infection had the potential to spread across the population.

**Table 4:** SIR model parameters and R0 values for Delhi: first wave Vs second wave.

Delhi	First wave	Second wave
Period	April 2020	April 2021
Cumulative total infected cases under two waves (end of April)	3515	27047
Maximum cases/per day under two waves (month of April)	356	25615
Contact rate, β	0.53	1.00
Recovery rate, γ	0.47	0.90
Reproductive number, R0	1.13	1.11

### 3.7 Pattern spread of COVID-19: first wave vs second wave

**Figure 6:**
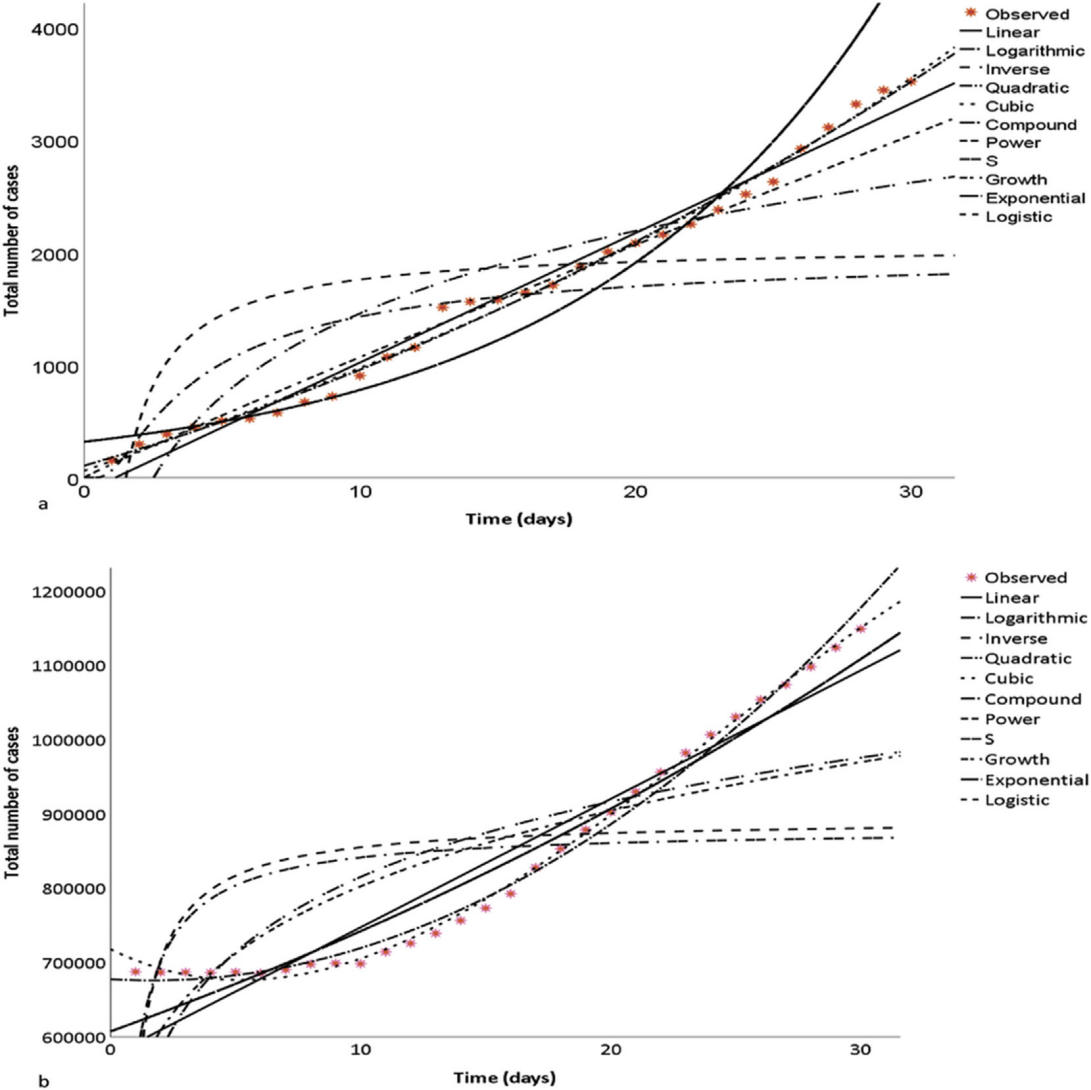
Curve fitting under different models for the observed number of COVID-19 cumulative cases for (a) April 2020 and (b) April 2021.

Curve fitting using the time series data of total cumulative confirmed COVID-19 cases in Delhi for April 2020 and April 2021 sheds light on the infection spreading trajectory of the successive waves. The curve fitting regression analyses for 11 different models and their agreements with the observed curve for total cumulative COVID-19 cases in Delhi for two periods, April 2020 and April 2021, are shown in Figure 6a and b, respectively. Table S3 summarises the parameter estimates for each of the 11 curves for the two different time frames. For April 2020 and April 2021, the cubic model $y=b_{0}+\left(b_{1}x\right)+\left(b_{2}x^{2}\right)+\left(b_{3}x^{3}\right)$ provides the best fit with the highest R^2^ values of 0.992 and 0.998 and the lowest standard errors of 94.19 and 0.998, respectively. The results suggest that the growth of COVID-19 cumulative cases did not take the exponential form in both waves of the COVID-19 pandemic in Delhi. Following the exponential growth model, the virus infection spread would have been larger and faster than under the cubic growth trajectory indicated in this study. In an exponential growth situation, the start of infection may be small; however, it would eventually have overtaken the growth of the cubic model by doubling with time. The spread of viral infection in real-life situations tends to follow exponential growth. However, the cubic growth trajectory indicated here shows the possible effect of the strict lockdown in the first wave and vaccination plus selective lockdown combination in the second wave, thus reducing the true virulence of the COVID-19 pandemic in Delhi.

## 4 Conclusions

This study concluded that the first wave unfolded in the city with three successive peaks and valleys, and the tallest peak was the third one, while the first peak of the second wave was the tallest. The doubling time for COVID-19 infected cases was shorter for the second wave than for the first wave.

This study found that the first wave of COVID-19 new cases and deaths were significantly and negatively correlated with the RH of Delhi. The ambient temperature of the first wave strongly and positively correlated with COVID-19 total cases primarily due to the COVID-19 super-spreading event reported in Delhi during March–April 2020. The study indicated that ambient PM_2.5_ and PM_10_ had no association with COVID-19 morbidity/mortality during either wave. A significant negative correlation was maintained between NO_2_ and COVID-19 (cases per day) even as the NO_2_ concentration increased during the second wave. During both waves, the ground-level O_3_ concentration had a significant positive association with COVID-19 mortality. These results indicate a possible virucidal role of SO_2_ during the first wave. The R0 of the two successive COVID-19 waves had only marginal differences, while the spread of COVID-19 new cases followed a cubic growth trajectory during both waves.

Despite presenting new insights about the impact of environmental variables across two successive COVID-19 waves, our study had limitations. First, this study used only limited meteorological variables. Second, the study did not consider the effect of confounding factors such as population density, socioeconomic factors, and virus mutation on SARS-COV-2 virus transmission. Third, Spearman's correlation coefficients did not show any causal relationships among the variables, although they were robust in showing a linear relationship.

## Declarations

### Author contribution statement

Abhishek Dutta: Conceived and designed the experiments; Performed the experiments; Analyzed and interpreted the data; Wrote the paper.

Gautam Dutta: Analyzed and interpreted the data; Contributed reagents, materials, analysis tools or data; Wrote the paper.

### Funding statement

This research did not receive any specific grant from funding agencies in the public, commercial, or not-for-profit sectors.

### Data availability statement

Data associated with this study has been deposited at http://health.delhigovt.nic.in/wps/wcm/connect/DoIT_Health/health/home/.

### Declaration of interests statement

The authors declare no conflict of interest.

### Additional information

No additional information is available for this paper.
