# Supplementary material for: Association of air pollution and meteorological variables with the two waves of COVID-19 pandemic in Delhi: A critical analysis
Source: Heliyon. 2021 Nov 24;7(11):e08468. doi: 10.1016/j.heliyon.2021.e08468 (PMC8610833; doi:10.1016/j.heliyon.2021.e08468)
Supplement: Supplementary materials [file mmc1.docx]

**Association of air pollution and meteorological variables with the two waves of COVID-19 pandemic in Delhi: A critical analysis**

**Abhishek Dutta ^^[[1]](#footnote-1)^^, Gautam Dutta ^b,*^**

^a^ Department of Environmental Science, Faculty of Science, Chulalongkorn University, 254 Phayathai Road, Pathumwan, Bangkok 10330, Thailand.

^b^ Department of Management Studies, Indian Institute of Foreign Trade, 1583, Madurdaha, Kolkata, West Bengal 700100, India.

**Table S1**

Metadata of the air pollution monitoring and weather stations

| **Name of the stations** | **Longitude East** | **Latitude North** | **Elevation above sea level (m)** |
| --- | --- | --- | --- |
| **Air pollution data** |  |  |  |
| Jahangirpuri | 77.1666° | 28.7296° | 211 |
| Patparganj | 77.3046° | 28.6347° | 214 |
| Jawahar Lal Nehru Stadium | 77.2344° | 28.5828° | 219 |
| Siri fort | 77.2148° | 28.5506° | 216 |
| **Weather data** |  |  |  |
| Indira Gandhi International Airport station | 77.10° | 28.57° | 236 |
| Vasant Vihar station | 77.1617° | 28.5603° | 214 |

**Table S2**

11 Classical regression models used for curve fitting.

| **SL No** | **Equations** | **Models** |
| --- | --- | --- |
| 1 | $y=b_{0}+b_{1}x$ | Linear model |
| 2 | $y=b_{0}+(b_{1}*\ln\left( x \right))$ | Logarithmic model |
| 3 | $y=b_{0}+(\frac{b_{1}}{x})$ | Inverse model |
| 4 | $y=b_{0}+\left( b_{1}x \right)+(b_{2}x^{2})$ | Quadratic model |
| 5 | $y=b_{0}+\left( b_{1}x \right)+\left( b_{2}x^{2} \right)+(b_{3}x^{3})$ | Cubic model |
| 6 | $y=b_{0}\left( x^{b_{1}} \right) or\ln\left( y \right)=\ln\left( b_{0} \right)+(b_{1}\ln\left( x \right))$ | Power model |
| 7 | $y=b_{0}\left( {b_{1}}^{x} \right) or\ln\left( y \right)=\ln\left( b_{0} \right)+(\ln\left( b_{1} \right)x)$ | Compound model |
| 8 | $y=e^{{(b}_{0}+(\frac{b_{1}}{x}))}$ | S-curve model |
| 9 | $y=\frac{1}{(\frac{1}{u}+\left( b_{0}\left( {b_{1}}^{x} \right) \right))}or\ln\left( \frac{1}{y}-\frac{1}{u} \right)=\ln\left( b_{0} \right)+(\ln\left( b_{1} \right)x)$, u denotes upper boundary value | Logistic model |
| 10 | $y=e^{{(b}_{0}+(b_{1}x))}or\ln\left( y \right)=b_{0}+{(b}_{1}x)$ | Growth model |
| 11 | $y=b_{0}\left( e^{\left( b_{1}t \right)} \right) or\ln\left( y \right)=\ln\left( b_{0} \right)+(b_{1}x)$ | Exponential model |

**Table S3**

Parameter estimates of 11 growth model curves under two different time frames of two COVID-19 waves, Delhi.

| Growth Equation | Model summary | | | | | Parameter estimation | |
| --- | --- | --- | --- | --- | --- | --- | --- |
| **April 2020** | **R^2^** | **F** | **df1** | **df2** | **p-value** | **Constants** | **Standard error** |
| Linear | 0.983 | 1627.387 | 1 | 28 | ≤ 0.05 | -130.107 | 135.177 |
| Logarithmic | 0.776 | 96.771 | 1 | 28 | ≤ 0.05 | -979.881 | 492.373 |
| Inverse | 0.353 | -15.253 | 1 | 28 | ≤ 0.05 | 18382243.894 | 836264 |
| Quadratic | 0.992 | 1711. 349 | 1 | 28 | ≤ 0.05 | -9188707.967 | 93.64 |
| Cubic | 0.992 | 1127.829 | 1 | 28 | ≤ 0.05 | -6125268.872 | 94.19 |
| Compound | 0.921 | 326.874 | 1 | 28 | ≤ 0.05 | 0.000 | 0.235 |
| Power | 0.971 | 922.978 | 1 | 28 | ≤ 0.05 | 0.000 | 0.144 |
| S model | 0.653 | 52.675 | 1 | 28 | ≤ 0.05 | 14334.743 | 0.493 |
| Growth | 0.921 | 326.874 | 1 | 28 | ≤ 0.05 | -14320.270 | 0.235 |
| Exponential | 0.921 | 326.874 | 1 | 28 | ≤ 0.05 | 0.000 | 0.235 |
| Logistic | 0.921 | 326.874 | 1 | 28 | ≤ 0.05 | 0.003 | 0.235 |
| **April 2021** |  | | | | | | |
| Linear | 0.920 | 321.007 | 1 | 28 | ≤ 0.05 | 574498.949 | 45773.879 |
| Logarithmic | 0.614 | 44.546 | 1 | 28 | ≤ 0.05 | 478512.871 | 100398.761 |
| Inverse | 0.217 | 7.763 | 1 | 28 | ≤ 0.05 | 893685.302 | 142993.722 |
| Quadratic | 0.991 | 1550.464 | 2 | 27 | ≤ 0.05 | 677746.660 | 15289.973 |
| Cubic | 0.998 | 3859.463 | 3 | 26 | ≤ 0.05 | 718099.466 | 0.998 |
| Compound | 0.938 | 421.267 | 1 | 28 | ≤ 0.05 | 607388.958 | 0.046 |
| Power | 0.646 | 51.061 | 1 | 28 | ≤ 0.05 | 539811.495 | 0.11 |
| S-curve | 0.235 | 8.608 | 1 | 28 | ≤ 0.05 | 13.689 | 0.162 |
| Growth | 0.938 | 421.267 | 1 | 28 | ≤ 0.05 | 13.317 | 0.046 |
| Exponential | 0.938 | 421.267 | 1 | 28 | ≤ 0.05 | 607388.958 | 0.046 |
| Logistic | 0.938 | 421.267 | 1 | 28 | ≤ 0.05 | 1.646E-06 | 0.938 |

|  |
| --- |
|  |

**Fig. S1.** AQI for Delhi during (a) April 2020 and (b) April 2021.

| \| 0-50 \| 51-100 \| 101-200 \| 201-300 \| 301-400 \| >401 \| \| --- \| --- \| --- \| --- \| --- \| --- \| \| 0% \| 53.33% \| 46.67% \| 0% \| 0% \| 0% \| | \| 0-50 \| 51-100 \| 101-200 \| 201-300 \| 301-400 \| >401 \| \| --- \| --- \| --- \| --- \| --- \| --- \| \| 0% \| 6.67% \| 66.67% \| 13.33% \| 13.33% \| 0% \| |
| --- | --- | --- | --- | --- | --- | --- | --- | --- | --- | --- | --- | --- | --- | --- | --- | --- | --- | --- | --- | --- | --- | --- | --- | --- | --- |

**Fig. S2.** Six AQI classes of Delhi under two waves (a) April 2020 (b) April 2021.

|  |  |
| --- | --- |
|  |  |
|  |  |
|  |  |
|  |  |
|  |  |

**Fig. S3**. Time series of criteria pollutants, meteorological variables and confirmed COVID-19 cases per day of Delhi (a) RH%, 2020 (b) RH%, 2021(c) Temperature, 2020 (d) Temperature, 2021 (e) NO_2_, April 2020 (f) NO_2_, April 2021 (g) Ozone , April 2020 (h) Ozone, April, 2021 (i) SO_2_, April 2020 (j) SO_2_ April 2021 (k) Windspeed, April 2020 and (l) Windspeed, April 2021.

1. *Corresponding author:

   *Email address*: gautam@iift.edu [↑](#footnote-ref-1)
